# Antimicrobial Usage in Commercial and Domestic Poultry Farming in Two Communities in the Ashanti Region of Ghana

**DOI:** 10.3390/antibiotics10070800

**Published:** 2021-06-30

**Authors:** Ellis Kobina Paintsil, Linda Aurelia Ofori, Charity Wiafe Akenten, Dennis Fosu, Seth Ofori, Maike Lamshöft, Jürgen May, Kwasi Obiri Danso, Ralf Krumkamp, Denise Dekker

**Affiliations:** 1 Kumasi Centre for Collaborative Research in Tropical Medicine (KCCR), South-End, Asuogya Road, Kumasi 039-5028, Ghana; danquah@kccr.de (C.W.A.); d.fosu@kccr.de (D.F.); sethofori@knust.edu.gh (S.O.); 2 Department of Theoretical and Applied Biology, Kwame Nkrumah University of Science and Technology, Kumasi 039-5028, Ghana; laandoh.cos@knust.edu.gh (L.A.O.); obirid@knust.edu.gh (K.O.D.); 3 Department Infectious Disease Epidemiology, Bernhard Nocht Institute for Tropical Medicine (BNITM), Bernhard-Nocht-Str. 74, 20359 Hamburg, Germany; lamshoeft@bnitm.de (M.L.); may@bni-hamburg.de (J.M.); krumkamp@bnitm.de (R.K.); dekker@bnitm.de (D.D.); 4 German Centre for Infection Research (DZIF), Partner Site Hamburg-Lübeck-Borstel-Riems, 20359 Hamburg, Germany; 5 Tropical Medicine II, University Medical Center Hamburg-Eppendorf (UKE), 20251 Hamburg, Germany

**Keywords:** antimicrobial resistance, antibiotics, poultry, commercial farms, domestic, free-range, herbs, veterinary officer

## Abstract

Poultry farming is a common practice in Ghana. Antibiotics are used, particularly in commercial poultry farming, as growth promoters and to prevent and cure infections. However, there is little information on antimicrobial usage in domestic poultry farming in Ghana. This study aimed to describe antimicrobial usage in commercial and domestic poultry farming. A cross-sectional survey was conducted within the Ashanti region of Ghana including 33 commercial farms and 130 households with domestic poultry farming. The median poultry population on commercial farms was 1500 (IQR: 300–3000) compared with 18 (IQR: 10–25) on domestic farms. The majority (97%, *n* = 32) of commercial farms used antimicrobials, compared with 43% (*n* = 56) of the domestic farms. Commercial farmers were 6.1 (CI: 3.2–11.8) times more likely to read and follow instructions on antimicrobials in comparison with domestic poultry keepers. About 11% of domestic and 34% of commercial farmers had received education on antimicrobial usage. None of the commercial farmers used herbal remedies; however, 40% (n/N = 52/130) of domestic farmers administered herbs. The misuse of antimicrobials in domestic poultry production calls for stricter regulations and training to limit the emergence and spread of antimicrobial-resistant bacteria among poultry.

## 1. Introduction

Domestic poultry farming considerably improves the socioeconomic status of families and communities in low- and middle-income countries [1]. In Ghana, most rural and semi-urban households own poultry [2]. In total, households own approximately 40 million poultry compared with an estimated 70 million kept on commercial farms [3]. Both commercial farms and free-range poultry production by households face challenges such as poor husbandry conditions, feeding status and diseases among farm animals [4]. To address some of these challenges, antimicrobials have been used for more than seven decades in poultry production as growth promoters and to prevent and treat infections [5,6,7].

Extensive use of antimicrobials in animals has contributed to an increase in antimicrobial resistance (AMR) [8]. Subsequently, the spread of multidrug-resistant bacteria has emerged as a global health threat [8,9]. AMR hampers the treatment of life-threatening infections in both humans and animals. Over the years, high numbers of antimicrobial-resistant bacteria have been isolated from patient samples of rural, urban and semi-urban health facilities in Ghana [10,11] as well as from locally produced and imported poultry [12]. In North Central Nigeria, it was observed that even though many commercial poultry farmers used antimicrobials, their level of knowledge regarding antimicrobial use (AMU) was low [13].

Few studies have been carried out in Ghana to describe antimicrobial usage in commercial poultry farms [14,15,16]. A previous study aimed at investigating essential antibiotics used in poultry production in Ghana found tetracyclines (24.17%), aminoglycosides (17.87%), penicillins (16.51%) and fluoroquinolones to be commonly administered [14]. Antibiotic residues have been found in the albumen and yolk of poultry eggs from farms that overly used antibiotics to cover up hygiene lapses [15]. About 41% of animal husbandry farmers in Ghana use antibiotics for infection prevention at least once a month [16]. Unlike commercial farms, no previous studies can be found regarding antimicrobial usage in domestic poultry in Ghana. However, this information is crucial for the development of interventions to reduce the spread of antimicrobial resistance. This study aims to investigate antimicrobial usage in both commercial and domestic poultry farming in two districts in the Ashanti region of Ghana.

## 2. Results

### 2.1. Frequencies of Poultry Produced by Commercial and Domestic Farmers

A total of 33 commercial poultry farms were included in this study, comprising 13 (39%) from Agogo and 20 (61%) from Ejisu. On the domestic farm level, 130 surveys were conducted including 70 (54%) from Agogo and 60 (46%) from Ejisu. The median commercial poultry population was 1500 (IQR: 300–3000) fowls, which was higher compared with the number of fowls kept by domestic farmers (median = 18; IQR: 10–26). Out of the 33 commercial farms visited by the study team, only two (6%, n/N = 2/33) were owned by females, but 55% (n/N = 72/130) of the domestic free-range poultry were owned by females.

### 2.2. Antimicrobial Use in Commercial and Domestic Poultry Farming

Details of antimicrobial usage patterns among commercial and domestic poultry farmers are given in Table 1. Most commercial poultry farmers (97%, n/N = 32/33) and 43% (n/N = 56/130) of the surveyed domestic farmers reported the use of antimicrobials. The majority of commercial farmers who used antimicrobials reported weekly administration (55%, n/N = 18/33), while almost half of the domestic poultry farmers administered it on a daily basis (45%, n/N = 25/56). Only 34% (n/N = 11/32) of the commercial and 10% (n/N = 6/56) of the domestic farmers reported having attended training on antibiotic usage in farming. In Ejisu, 55% (n/N = 33/60) of domestic poultry owners administered antimicrobials compared with 32.9% (n/N = 23/70) in Agogo (PR = 1.7, 95%-CI: 1.1–2.5). The percentage usage of antimicrobials at farms was 100% (n/N = 20/20) in Ejisu and 92.3% (n/N = 12/13) in Agogo.

A veterinary officer had visited and provided various services to 53% (n/N = 17/32) of commercial farmers that used antimicrobials compared with 11% (n/N = 6/56) of domestic poultry farmers during the last 12 months (PR = 5.0, 95%-CI: 2.2–11.3). The services of veterinary officers were 2.5 (95%-CI: 1.1–5.9) times more likely to be demanded by a commercial farmer than by a domestic farmer. In the case of an infectious disease outbreak, veterinary officers visited more commercial farms than domestic poultry farms (PR = 6.1, 95%-CI: 1.4–27.7). Furthermore, commercial poultry farmers were 6.1 (95%-CI: 3.2–11.8) times more likely to read instructions on antimicrobials compared with domestic poultry keepers. Also, commercial farmers were 3.2 (95%-CI: 1.3–7.9) times more likely to report using antimicrobials as growth promoters than households with domestic poultry. None of the commercial farmers used herbal remedies; however, 40% (n/N = 52/130) of households with free-range domestic fowls administered herbs. Most (82%, n/N = 46/52) of them applied *Tetrapleura tetraptera* (locally called prekese), and 62% (n/N = 32/52) applied the bark of mango trees (*Mangifera indica*).

Table 2 summarizes factors associated with antimicrobial use on the surveyed farms. Crude PRs and PRs adjusted by Poisson regression are given in the table. The regression analysis shows that farmers working on commercial poultry farms and living in a semi-urban environment (i.e., Ejisu) and farmers who use herbal medicines are more likely to give antimicrobials to their animals. The gender of the farm manager and a visit from a veterinary officer during the last year did not show any association with antibiotic usage.

### 2.3. Antimicrobial Active Ingredients

**Figure 1:**
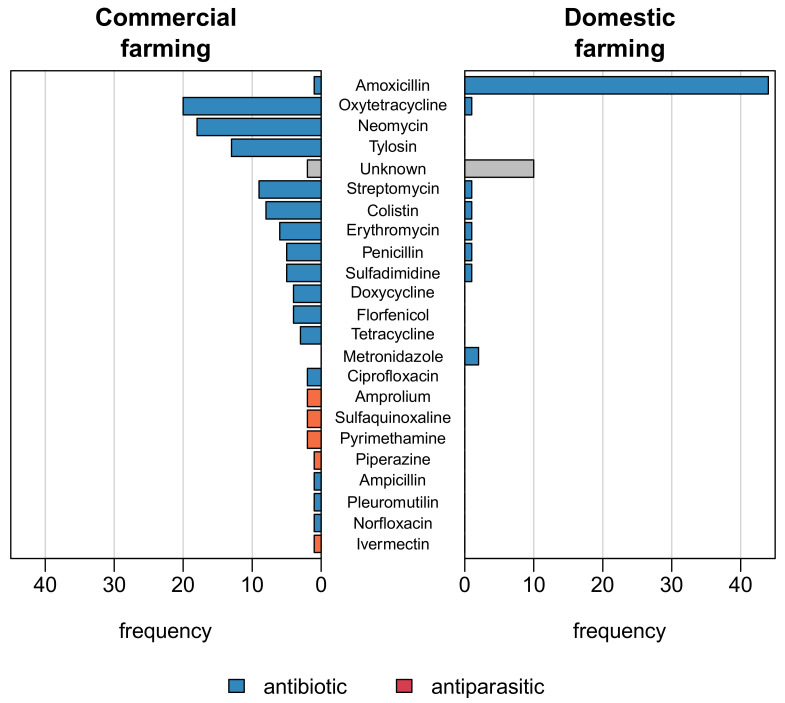
Types of antimicrobial active ingredients employed by commercial and domestic farmers.

Figure 1 shows the frequencies and the different types of antimicrobial active ingredients employed by commercial and domestic poultry farmers. In total, commercial poultry farmers used 21 different antimicrobial active ingredients belonging to 11 classes. Five of the active ingredients were antiparasitics, and the rest (16) were antibiotics. The majority (62.5%, n/N = 20/32) of commercial farmers administered antimicrobials with at least three active ingredients compared with one active ingredient being administered by 80.4% (n/N = 45/56) of domestic poultry keepers. Oxytetracycline (62.5%, n/N = 20/32), neomycin (56.2%, n/N = 18/32), tylosin (40.6%, n/N = 13/32), streptomycin (28.1%, n/N = 9/32) and colistin (25.0%, n/N = 8/32) were the common active ingredients contained in the antimicrobials employed by the commercial poultry farmers. In contrast, eight different active ingredients were employed by the 56 domestic poultry farmers; however, 44 (78.6%) of them administered only amoxicillin. 

## 3. Discussion

We report that almost half (43.1%) of domestic poultry farmers in the study area used antimicrobials in animal rearing, which is more than 6 times higher than data reported from Nigeria (7.9%) [13]. The strikingly high frequencies of antimicrobial usage shown in the current study could be due to knowledge differences in study populations and a generally increasing availability of antimicrobials. Also, almost all (97.0%) of the commercial poultry farms used antimicrobials, which is in agreement with studies conducted in Bangladesh (98%) [17], Nepal (90%) [18] and Nigeria (89%) [19]. The high prevalence of AMU could be attributed to administering antimicrobials for prophylaxis and growth promotion purposes. Our findings are of public health importance because excessive use (or misuse) of antimicrobials in poultry production will inevitably lead to the transmission of resistant bacteria to humans and the environment [8,9].

We observed that overuse or misuse of antimicrobials was more common in large-scale farming. A possible explanation for this is that the sheer number of animals kept in a confined space may also favor the transmission of infections, which makes prophylactic administration of antimicrobials more likely. However, unlike commercial farmers, domestic poultry owners typically reported using antimicrobials to treat infections and less for promoting growth. This could be because domestic poultry is free-roaming and because owners are less profit-oriented [20] and lack knowledge on the various types of antimicrobials and their application. Farmers should be encouraged to improve hygiene and vaccine administration and implement biosecurity measures since this has been shown to increase production in the absence of AMU [21,22].

Even though a lower number of antimicrobials was used in domestic poultry compared with commercial poultry farming, domestic farmers showed a higher risk for inappropriate antimicrobial usage. This study observed that about half (45%) of domestic farmers who used antimicrobials administered them daily, which is an alarming finding. The daily routine usage of antimicrobials in domestic poultry increases the risk of antimicrobial-resistant bacteria [23,24,25]. In addition, this practice puts people at risk of ingesting antimicrobial residues through food and drinking water. These findings are supported by Alhaji et al. [13] who also reported improper antimicrobial use in local bird flocks. This study noted that 52% of domestic farmers who administered antimicrobials used those that were recommended by friends and easily acquired them from the nearest pharmacy stores. The easy access to antimicrobials may be a reason for not seeking the professional services of a veterinarian and another reason why domestic poultry owners lack adequate information on the correct usage and dosage of antimicrobials [13].

This study found that veterinary officers primarily visited commercial poultry farms or domestic poultry households when their services were demanded. Domestic poultry farmers are often unable to afford the services of veterinary officers; therefore, they were more in favor of using herbs for prophylaxis and infection treatment as shown by the current study and by other studies [26,27]. In addition, domestic farmers who applied herbal treatments were more likely to also use antimicrobials. The use of antimicrobials to treat infections in the poultry industry has economic and productivity benefits [28,29]. However, to reap these benefits, veterinary officers should restrict access by reducing their antimicrobial prescriptions and should educate farmers on proper drug administration. 

Almost all domestic farmers who employed antibiotics used only amoxicillin; however, commercial farms employed antimicrobials with at least three different types of active ingredients, the most common ones being oxytetracycline, tylosin, streptomycin, neomycin and colistin. Previous studies have also reported on the use of these antimicrobials in poultry farming [30,31,32]. We, therefore, conclude that poultry farming might be an important but underestimated source of transmission of antimicrobial-resistant bacteria in Ghana. Despite the awareness that animal and human data are relevant to monitor the current spread of antimicrobial-resistant bacteria, One Heath surveillance systems that link the different data sources are still vastly missing in Ghana. However, such systems are essential to monitor and control the use of antibiotics and the spread of AMR from animal farming.

Our study has limitations that should be considered when interpreting the data. We collected data from only two communities (rural and semi-urban) in the Ashanti region, Ghana. To generalize our findings, data from other communities and regions would be needed. Antimicrobial usage behavior of commercial and domestic poultry farmers could be influenced by age, years of farming experience and educational level, which were not considered in our study. However, such data would be crucial to develop targeted training programs for the proper use of antimicrobials in poultry farming.

## 4. Materials and Methods

### 4.1. Study Areas

**Figure 2:**
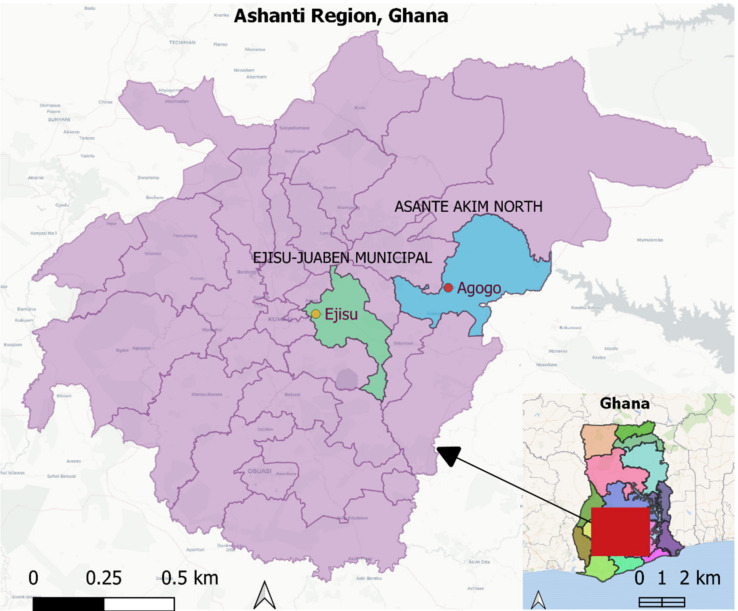
Geographical location of Agogo and Ejisu in the Ashanti region, Ghana.

The study was conducted in Agogo and Ejisu, located within two districts in the Ashanti region of Ghana (Figure 2) [33]. Agogo, the capital of the Asante Akim North municipality, is a rural community with a projected population of 94,297 [34]. It is located in the eastern part of the Ashanti region, 82 km by road from Kumasi, the capital city of the Ashanti region. Most households of the Asante Akim North municipality (98%) are involved in crop farming [34]. Chicken is reared by 42% of the population and accounted for 56% of livestock kept in the district [34]. 

Ejisu is a semi-urban community located within the Ejisu Juaben municipality. It is the fifth-largest district in the Ashanti region, occupying a total land area of 582 square kilometers. The current projected population is 188,628 with 57% of households engaged in agriculture. About 21% of the 23,416 households in the municipality rear livestock, and poultry accounts for 70% of the livestock [34]. 

### 4.2. Study Design

A cross-sectional survey was conducted among commercial poultry farms and households that kept domestic chickens in rural (Agogo) and semi-urban (Ejisu) communities in Ghana. Interviews were conducted between January and October 2020. We developed, piloted and validated a questionnaire with fourteen items, 2 open and 10 close-ended questions. The final questionnaire included: two questions on participants’ characteristics, two questions on the type and quantity of poultry kept, four questions on antimicrobial administration, and six questions on veterinary services offered to the farm. Two of the close-ended questions were “yes/no” questions; the remaining were multiple-response questions.

The questionnaire was administered to 13 farms and 70 households in the Agogo community. In Ejisu, it was administered to 20 farms and 60 households; the same questions were asked in both the farms and households. All farms and households were selected using snowball sampling. From each community, one or two poultry farms and potential households rearing free-range poultry were initially identified, and we requested that they introduce us to another poultry farm or household for possible sampling. At the commercial farm level, we interviewed the farm managers, and at the household level, we interviewed the poultry owners. A trained study team member carried out the interviews and inspected the packaging of currently used antimicrobial medications to obtain data on the active agents.

### 4.3. Data Analysis

The median along with the interquartile range (IQR) was used to summarize the poultry population. Absolute frequencies and their corresponding percentages were used to describe categorical variables. Crude prevalence ratios (PR) and their corresponding 95% confidence intervals (CI) were calculated to show associations between two categorical variables. Poisson regression, with robust standard errors, was calculated considering all variables of interest to control for potential confounding. There were no missing data; due to the explanatory nature of the study, no significance testing was applied. All statistical analyses were performed using *R* (version 4.0.3, R Core Team, Vienna, Austria) software [35]; the *epiR* (version 2.0.19, R Core Team, Vienna, Austria) package was used to calculate PRs, and the *sandwich* package (version 3.0-0, R Core Team, Vienna, Austria) was used to compute robust estimators of the Poisson regression.

## 5. Conclusions

This study revealed high levels of antimicrobial usage in both commercial and domestic poultry farming, with a potential impact on One Health. Therefore, interventions such as tailored antimicrobial stewardship programs, improved hygiene, biosecurity and vaccination programs should be considered to control antimicrobial use in poultry farming. Our findings call for the development of stricter regulations on access and usage of antimicrobials in both domestic and commercial poultry as well as an improvement in the surveillance of antimicrobial-resistant bacteria.

## Figures and Tables

**Table 1:** Antimicrobial usage in commercial farms and domestic poultry, Ashanti region, Ghana.

Variables	Commercial Farms (N = 33) n (%)	Domestic Farms (N = 130) n (%)
Frequency of antimicrobial administration		
Daily	1 (3)	25 (19)
Weekly	18 (55)	6 (5)
Monthly	6 (18)	4 (3)
1–3 months	3 (9)	15 (12)
Only when infection occurs	4 (12)	6 (2)
Never	1 (3)	74 (57)
Reasons for administering antimicrobials		
To promote growth	11(35)	6 (11)
Prophylaxis	30 (94)	37 (66)
To treat infections	8 (25)	36 (64)
Source of antimicrobial supply		
Pharmacy store	6 (19)	50 (89)
Veterinary store	26 (81)	6 (11)
Who introduced you to the antimicrobial		
Veterinary Officer	18 (56)	6 (11)
Pharmacy store	1 (3)	5 (9)
Friends	8 (25)	29 (52)
Others	5 (16)	16 (29)
Follow instructions	28 (88)	8 (14)
Educated on antimicrobial administration	11 (34)	6 (11)

**Table 2:** Associations with probability of using antimicrobials in poultry farming, Ashanti region, Ghana.

Variables	Crude Ratio PR (95%-CI)	Adjusted Ratio PR (95%-CI)
Commercial farm (vs. domestic farm)	2.3 (1.8–2.8)	2.9 (1.9–4.3)
Semi-urban area (Ejisu vs. Agogo)	1.5 (1.2–2.1)	1.3 (1.0–1.8)
Sex (female vs. male)	0.7 (0.5–1.0)	1.0 (0.7–1.4)
Veterinary officer visit during the last year	1.4 (1.1–1.9)	1.1 (0.8–1.4)
Herbal medicine used	1.2 (0.9–1.6)	1.9 (1.3–2.8)

## Data Availability

The data used to support the findings of this study are available from the corresponding author upon request.
